# Evaluation of In Vivo Efficacy and In Vitro Shear Bond Strength of Fluoride-Releasing Orthodontic Composite and Fluoride Varnish

**DOI:** 10.7759/cureus.77823

**Published:** 2025-01-22

**Authors:** Naman Mittal, Rajat Mangla, Deepankar Bhatnagar, Mandeep K Bhullar, Gaurav Ahuja, Arti Devi

**Affiliations:** 1 Orthodontics and Dentofacial Orthopedics, Maharishi Markandeshwar College of Dental Science and Research, Ambala, IND; 2 Orthodontics, Mangla Orthodontic and Dental Care, Jagadhri, IND

**Keywords:** demineralization index, fluoride-releasing orthodontic composite, fluoride varnish, gingival index, plaque index, shear bond strength

## Abstract

Introduction: The fluoride varnish and fluoride-releasing composites are being used in orthodontic patients to prevent white spot lesions (WSL). The study evaluates and compares the efficacy of these two on plaque accumulation, gingival health, and demineralization in vivo, as well as shear bond strength in vitro.

Methods: This study was conducted using 45 extracted premolars for the in vitro group and 45 patients for the in vivo group. Each group was further subdivided into three subgroups of 15 each. In the in vitro subgroups, bonding in Subgroup I (control group) was done using Transbond XT, Subgroup II (fluoride varnish group) involved the application of fluoride varnish using Fluor Protector, and Subgroup III (Transbond Plus Color Change Adhesive) was used for bonding. Shear bond strength was evaluated in the in vitro group using a universal testing machine. In the three in vivo subgroups, bonding was done similarly to the in vitro group. The efficacy was assessed in vivo using the plaque index (PI), gingival index (GI), and demineralization index at T0 (baseline), T1 (week 4), T2 (week 8), and T3 (week 12).

Results: The results demonstrated a statistically significant difference between the inter- and intragroup comparisons of the PI and GI, whereas the demineralization index group did not show any statistically significant difference, as indicated by ANOVA, Tukey’s test, and paired t-tests. The in vitro group showed statistically significant differences between the fluoride varnish group and both the control group and the fluoridated composite group.

Conclusion: Fluoride-releasing orthodontic composite and fluoridated varnish can be used to prevent plaque accumulation and demineralization and maintain gingival health during fixed orthodontic therapy.

## Introduction

White spot lesions (WSL) are formed when there is compromised oral hygiene during fixed orthodontic treatment because of the presence of various components like brackets, ligature wires, bands, and other orthodontic appliances, which are attached. This leads to an increase in plaque formation and increase in microbial activity, which forms an acidic environment causing a decrease in the pH of the saliva. This decrease in pH consequently causes a reduction in the concentration of fluoride and the movement of Ca²⁺ and PO₄³⁻ from the enamel apatite into the solution. Porosities are formed on the enamel surface from which micro-organisms penetrate into the sub-surface layers due to which WSL are formed [1].

The use of fluoride varnish over other topical fluoride-releasing products is recommended because studies have shown that it is more effective in increasing fluoride uptake in enamel. Additionally, fluoride varnish is easier to apply. Sodium fluoride is the most commonly used agent in fluoride varnish for preventing the formation of WSL [2].

Transbond Plus Color Change Adhesive (3M Unitek) is a material that contains fluorosilicate glass as the source of fluoride. The material is hydrophilic, which allows the diffusion of fluoride from within the cross-linked cured matrix in an aqueous medium [3].

When fluoride is applied to the tooth, it is deposited in hydroxyapatite and forms fluorapatite, which may, in turn, affect shear bond strength. The topical application of fluoride can hinder effective etching by phosphoric acid, leading to a reduction in the shear bond strength of the dental resin [4]. Several factors, such as contamination with saliva, fluoride content from toothpaste, oils, and other agents, can also affect shear bond strength [5].

Thus, the aim of our study was to evaluate the efficacy of and compare the shear bond strength between fluoride varnish and fluoridated orthodontic composite.

## Materials and methods

This study was conducted in the Department of Orthodontics & Dentofacial Orthopedics, Maharishi Markandeshwar College of Dental Science and Research, Mullana, during the period from 15-12-2019 to 22-12-2021. The sample consisted of 45 patients undergoing fixed orthodontic therapy for the in vivo study and 45 freshly extracted human premolar teeth for the in vitro study. Prior to the start of the study, approval was obtained from the Institutional Ethics Committee (IEC) and the Board of Studies of Maharishi Markandeshwar Deemed to be University, Mullana, Ambala (IEC-1589).

The inclusion criteria for the in vivo group were patients aged between 12 and 30 years, in good overall health, and requiring at least six months of fixed orthodontic therapy. For the in vitro group, the criteria included sound teeth with normal anatomy, no developmental defects, non-carious teeth, no enamel cracks, and an intact enamel surface. The exclusion criteria for the in vivo group included hypocalcified teeth, active periodontal disease, vestibular caries, a history of previous orthodontic treatment, fluorosis, a positive pregnancy test, and any systemic illness. For the in vitro group, the exclusion criteria included proximal caries, occlusal caries, grossly decayed teeth, fluorosis or fractured crowns, and hypoplastic or aberrant teeth.

In the in vivo group, patients who met the inclusion criteria were selected and randomly divided into three groups.

Group 1 (control group)

In this group, the teeth were bonded with orthodontic brackets using the conventional orthodontic bonding procedure and Transbond XT (3M Unitek) adhesive.

Group II (fluoride varnish group)

In this group, the teeth were bonded using the conventional bonding procedure. After bonding, the teeth were dried, and a layer of Fluor Protector (Ivoclar Vivadent) was applied to the tooth surfaces of the maxillary and mandibular right and left lateral incisors, canines, and second premolars. The fluoride varnish was allowed to dry for five minutes, and patients were advised not to drink for 30 minutes, not to eat for 4 hours, and to avoid brushing their teeth until the next morning after the fluoride varnish application. Patients were prohibited from using any other fluoride supplements.

Group III (fluoridated composite group)

In this group, the teeth were bonded using the fluoride-releasing orthodontic composite. Oral hygiene instructions were given to all the patients. The evaluation of plaque index (PI), gingival index (GI), and decalcification (WSL) was performed at baseline, 4 weeks, 8 weeks, and 12 weeks.

The PI was adapted from Bock et al., as introduced by Attin et al. [6,7], where the scoring was done on a scale from 0 to 3: 0 indicating no visible plaque, 1 indicating moderate accumulation on surfaces lateral to the brackets, 2 indicating moderate accumulation on the surfaces lateral and cervical to the brackets (with islands of plaque cervical to the brackets), and 3 indicating moderate accumulation of plaque on surfaces lateral to the brackets and 1/3 of the surface gingival to the bracket covered with plaque.

GI was measured using the Loe and Silness GI [8], with scores ranging from 0 to 3: 0 - normal gingiva; 1 - mild inflammation with slight color change and slight edema, no bleeding on probing; 2 - moderate inflammation with redness, edema, and glazing, along with bleeding on probing; and 3 - severe inflammation with marked redness and edema, a tendency for spontaneous bleeding, and ulceration.

The demineralization index was adapted from Leonard Gorelick, Arnold M. Geiger, and A. John Gwinnett [9], with scores ranging from 1 to 4: 1 - no white spot formation; 2 - slight white spot formation; 3 - excessive white spot formation; and 4 - white spot formation with cavitation. The PI, GI, and demineralization index were calculated at T0 (baseline), T1 (week 4), T2 (week 8), and T3 (week 12). The extracted tooth samples were stored in distilled water or saline from the time of extraction until the bonding procedure was performed. The storage medium was changed regularly to prevent bacterial contamination. The samples were randomly divided into three groups, with 15 samples in each group. The samples were then mounted on self-cure acrylic, and the buccal surface of the teeth was kept exposed for bonding the orthodontic brackets.

The teeth were then polished and dried using an oil-free three-way syringe. After drying, the teeth were etched with 37% phosphoric acid on the buccal surface for 20-30 seconds, after which they were rinsed with water using the oil-free three-way syringe to achieve a frosty white appearance. Next, a bonding agent was applied to the teeth and cured for 20 seconds. Group I teeth were bonded with stainless steel brackets using Transbond XT, Group III teeth were bonded using Transbond Plus Color Change adhesive, and Group II teeth were treated with fluoride varnish six hours before bonding. After bonding, the teeth were immersed in water for 24 hours, after which they were subjected to shear bond strength testing using a universal testing machine. The load applied by the universal testing machine was recorded in Newtons (N) and converted into MPa by dividing the load by the mean area of the bracket bases. Paired t-tests were used for intragroup comparisons, and ANOVA with Tukey’s test was used for intergroup comparisons. We used Tukey’s test in conjunction with ANOVA to compare the means of all the groups. The significance level was set at p<0.05.

## Results

In vivo

Intergroup comparison of the PI shows a statistically significant difference at the 4-week and 8-week intervals, with significant differences between Group 1 vs. Group 2, Group 2 vs. Group 3, and Group 1 vs. Group 3 at 4 weeks, and between Group 1 vs. Group 2 and Group 2 vs. Group 3 at 8 weeks, based on the results of ANOVA and Tukey’s Test (p<0.05) (Table 1).

**Table 1:** Intergroup comparison of PI PI, plaque index

PI score	Group 1 (mean and SD)		Group 2 (mean and SD)		Group 3 (mean and SD)		F	P-value	Group 1 vs. Group 2 P-value	Group 1 vs. Group 3 P-value	Group 2 vs. Group 3 P-value
Baseline	0.56	0.16	0.53	0.12	0.53	0.18	0.197	0.822	0.877	0.828	0.995
4 week	2.31	0.18	1.50	0.17	1.72	0.15	92.517	0.001	0.001	0.001	0.002
8 week	2.27	0.20	1.85	0.15	2.08	0.18	21.752	0.001	0.001	0.011	0.003
12 week	2.27	0.14	2.22	0.14	2.34	0.14	2.707	0.078	0.585	0.389	0.064

Intergroup comparison of the GI between Group 1, Group 2, and Group 3 showed statistically significant differences at 8 weeks and 12 weeks, and between Group 1 vs. Group 2 and Group 2 vs. Group 3 at 4 weeks, 8 weeks, and 12 weeks, as shown by ANOVA and Tukey’s test (p<0.05) (Table 2).

**Table 2:** Intergroup comparison of GI GI, gingival index

GI	Group 1 (mean and SD)		Group 2 (mean and SD)		Group 3 (mean and SD)		F	P-value	Group 1 vs. Group 2 P-value	Group 1 vs. Group 3 P-value	Group 2 vs. Group 3 P-value
Baseline	0.21	0.23	0.12	0.19	0.04	0.08	3.060	0.057	0.370	0.046	0.515
4 week	1.22	0.27	0.25	0.37	0.39	0.46	29.138	0.001	0.001	0.001	0.545
8 week	1.43	0.29	0.59	0.37	0.72	0.47	20.391	0.001	0.001	0.001	0.615
12 week	1.83	0.34	0.72	0.44	0.96	0.37	34.382	0.001	0.001	0.001	0.232

Intragroup comparison of PI scores for Group 1, Group 2, and Group 3 showed statistically significant differences at 4, 8, and 12 weeks, as confirmed by the paired t-test (p<0.05). The intragroup comparison of GI scores for Group 1, Group 2, and Group 3 showed statistically significant differences at 4, 8, and 12 weeks, as shown by the paired t-test (p<0.05). Intergroup and intragroup comparisons of the demineralization index showed no significant differences between the groups over the course of time (Table 3).

**Table 3:** Intergroup comparison of demineralization index

Demineralization index	Group 1 (mean and SD)		Group 2 (mean and SD)		Group 3 (mean and SD)		F	P-value	Group 1 vs. Group 2 P-value	Group 1 vs. Group 3 P-value	Group 2 vs. Group 3 P-value
Baseline	1.00	0.00	1.00	0.00	1.00	0.00	0.000	1.000	1.000	1.000	1.000
4 Week	1.09	0.19	1.00	0.00	1.00	0.00	3.658	0.064	0.061	0.061	1.000
8 Week	1.02	0.06	1.00	0.00	1.00	0.00	2.154	0.129	0.183	0.183	1.000
12 Week	1.02	0.09	1.00	0.00	1.01	0.03	0.672	0.516	0.484	0.832	0.832

In vitro

Shear bond strength values of Group 1, Group 2, and Group 3 are presented. The results show a statistically significant difference between the fluoride varnish group and both the control group and the fluoridated composite group, as indicated by ANOVA and Tukey’s test (p<0.05) (Table 4).

**Table 4:** Mean and standard deviation of shear bond strength

Shear bond strength	Group 1		Group 2		Group 3		F	P-value	Group 1 vs. Group 2	Group 1 vs. Group 3	Group 2 vs. Group 3
	Mean	SD	Mean	SD	Mean	SD			P-value	P-value	P-value
Bond strength (MPa)	17.51	5.57	7.91	3.85	15.00	7.28	11.302	0.001	0.001	0.239	0.002

## Discussion

A significant milestone in orthodontic therapy was the introduction of enamel bonding for orthodontic appliances in 1964 [5]. Buonocore, Tavas, and Watts were the pioneers in developing direct bonding techniques for attaching brackets to enamel [10,11]. Over time, the bonding procedures in orthodontics were simplified, which contributed to improvements in clinical practice. However, demineralization and other side effects of fixed orthodontic treatment remained significant problems [12].

Plaque index

The PI used in this study was adapted from Bock et al. (2005), which was introduced by Attin et al. (2005) [6,7]. This PI was chosen for its ability to score areas of high risk around the brackets (Arici et al., 2007; Mizrahi, 1982; Mitchell, 1992; Chang et al., 1997) [13-15]. The disclosing agent (Alpha Plac) was used because it contains several coloring agents or dyes that stain the biofilms, thus aiding in the easy visualization of dental plaque accumulation [16].

The results of our study showed statistically significant differences in the PI. Although the PI scores increased progressively in all the groups, they were lowest in Group 2, followed by Group 3 and Group 1. These results indicate that fluoride varnish is more effective in controlling plaque and maintaining gingival health during fixed orthodontic therapy.

Several studies have shown that fluoride varnish has the ability to release fluoride for up to 28 weeks [17]. The topical application of fluoride varnish leads to the deposition of calcium fluoride on the enamel surface, which remains there and in biofilms for weeks. Humidity does not affect fluoride varnish, which is another reason the varnish stays on the teeth for a longer period of time [18].

Fluoride is actively released by fluoride-releasing adhesives for 60 to 90 days. Transbond Plus releases fluoride at a rate of about 500 µ/day/gm of material and decreases by approximately 0.0073 µ/cm²/d [19]. Since fluoride varnish requires only a short application time and less patient compliance, it can be applied by staff, making it more advantageous to use [20].

Gingival index

The GI used was adapted from Loe and Silness [8]. There was a statistically significant difference in GI both between the groups and within the groups.

The color-changing property of Transbond Plus facilitates better flash cleaning around the brackets, thereby reducing plaque-retentive surfaces, which in turn helps in maintaining gingival health [3]. The antimicrobial property of fluoride release also disrupts bacterial accumulation in plaque, thus aiding in the maintenance of gingival health [21]. The improvement in gingival health may also be due to the Hawthorne effect, which states that when patients are included in a study, they experience increased self-motivation, ultimately leading to positive effects on the maintenance of oral hygiene [22].

Demineralization index

The demineralization index was adapted from Leonard Gorelick, Arnold M. Geiger, and A. John Gwinnett. It was the first visual method that could be easily used to clinically evaluate demineralization during orthodontic therapy. Additionally, this index could be easily used to determine the incidence and prevalence of demineralization [9,22].

Fluoride varnish helps in the prevention of the formation of WSL, as the topical application of fluoride varnish causes fluoride to react with the calcium in the teeth, leading to the formation of calcium fluoride. This calcium fluoride is considered a reservoir of fluoride ions, which helps protect against demineralization. SEM studies conducted by Stookey and Petersson have shown a layer of varnish adhered to the bracket base; this layer protects the calcium fluoride from early dissolution, thereby prolonging the protective effect of the varnish [18,23,24].

Although various studies have reported the formation of WSL within 4 weeks of starting fixed orthodontic therapy, the non-significant difference in the demineralization index may be due to several factors. A small amount of adhesive is used for bonding the brackets to the teeth, which do not contain sufficient fluoride ions. Additionally, there is no interaction between saliva and resin, as the composite resin is hydrophobic in nature, and the curing of the composite using a light source reduces the fluoride ion release ability [20]. The formation of WSL may take time in the patients recruited for the study, and not all WSL lead to the formation of carious lesions [25].

The sensitivity of the indices used in this study to evaluate oral hygiene is questionable for detecting differences in oral hygiene maintenance, which can be considered a limitation of the current in vivo study. Additionally, small but clinically relevant plaque accumulations may not always be adequately defined by the plaque indices, and the use of digital plaque measurement is desirable [26].

Shear bond strength

The minimum bond strength required to withstand normal orthodontic forces is believed to be between 6 and 8 MPa (Reynolds, 1975) [27]. In the current study, all materials exhibited adequate bond strength. The results of various studies regarding shear bond strength with pretreatment fluoride varnish application have been controversial, as reported by studies conducted by Bishara et al., 1989, and Meng et al., 1997 [5,28]. The results of this study are consistent with those of Meng et al., 1997, and Cacciafesta et al., 2005, which showed that pretreatment fluoride varnish interferes with the acid etching process, resulting in decreased shear bond strength. The difference in the modes of application of topical fluoride, such as applying it to enamel surfaces that are etched versus applying varnish after bonding the brackets, also yields controversial results [28,29].

Studies conducted by Gwinnett and Smith showed that fluoride tag formation is disrupted by topical fluoride application, which in turn significantly reduces bond strength. The differences in results among various studies may be due to factors such as the timing of application, use of fluoride varnishes with different fluoride concentrations, retention mechanisms of the brackets used, and the use of bonding materials with improved properties [4,30]. The values obtained in Newtons were converted into MPa by dividing by the surface area of the bracket [30].

A limitation of the current study is that the evaluation period was limited to 24 hours, whereas the average orthodontic treatment duration is up to two years. Therefore, it is suggested that future research evaluate shear bond strength at intervals of at least six, 12, 18, and 24 months. Additionally, the inability to ensure the precise distribution of stress at the bracket-adhesive interface for each sample adds to the limitation of the in vitro part of this study.

## Conclusions

The results of our study showed in vivo that fluoride varnish was more effective in controlling plaque accumulation, maintaining gingival health, and preventing the formation of WSL, followed by the fluoride-releasing orthodontic composite group and the conventional bonding procedure group. In vitro, the bond strength of the fluoride varnish group was statistically significantly lower compared to the other two groups. However, the differences in shear bond strength values were smaller in teeth where fluoride varnish was applied, compared to the fluoride-releasing composite group and the conventional bonding group. Despite this, the fluoride varnish group still provided adequate bond strength for orthodontic bonding. Hence, the results of our study suggest that fluoride-releasing orthodontic composites and fluoride varnish can be used to prevent plaque accumulation and demineralization and maintain gingival health during fixed orthodontic therapy.

## Appendices

Maharishi Markandeshwar (Deemed to be) University, Mullana (Ambala) Informed Consent Form

Study Title: ___________________________________

Study Number: __________________________________

Subject’s Full Name: ____________________________________

Date of Birth/Age: ____________________________________

Address: ____________________________________________________________

1. I confirm that I have read and understood the information sheet dated ______________ for the above study and have had the opportunity to ask questions. OR I have been explained the nature of this study by the Investigator and have had the opportunity to ask questions.

2. I understand that my participation in this study is voluntary and that I am free to withdraw at any time, without giving any reason and without my medical care or legal rights being affected.

3. I understand that the investigator of the clinical trial, others working on the investigator’s behalf, the ethics committee, and the regulatory authorities will not need my permission to look at my health records, both in respect of the current study and future research that may be conducted in relation to it, even if I withdraw from the trial. However, I understand that my identity will not be revealed in any information released to third parties or published.

4. I agree not to restrict the use of any data or results that arise from this study, provided such use is only for scientific purposes.

5. I agree to take part in the above study.

Signature (or thumb impression) of the subject/Legally Acceptable Representative: ___________ Signatory’s Name: ___________________________ Date: _____________________

Signature of the Investigator: _______________________ Date: ________________________

Study Investigator’s Name: _________________________

Signature of the Witness: ___________________________

Name of the Witness: _____________________________
